# 右侧非小细胞肺癌患者双肺叶切除与单肺叶切除术后并发症的比较

**DOI:** 10.3779/j.issn.1009-3419.2014.08.03

**Published:** 2014-08-20

**Authors:** 颖 陈, 玉洁 雷, 云超 黄, 联华 叶, 光强 赵, 光剑 李, 凯云 杨, 秋博 黄

**Affiliations:** 650118 昆明，昆明医科大学第三附属医院，云南省肿瘤医院胸外科一病区 Deparment of Thoracic Surgery, the Third Affiliated Hospital of Kunming Medical University, Tumor Hospital of Yunnan Province, Kunming 650118, China

**Keywords:** 肺肿瘤, 双肺叶切除, 术后并发症, Lung neoplasms, Bilobectomy, Postoperative complications

## Abstract

**背景与目的:**

双肺叶切除后剩余肺叶与胸膜腔空间不匹配是右侧非小细胞肺癌(non-small cell lung cancer, NSCLC)患者术后高并发症发病率的主要原因。本研究旨在比较右侧NSCLC双肺叶切除与单肺叶切除患者术后并发症发生率之间的差异。

**方法:**

本研究共纳入行右侧肺叶切除NSCLC 528例, 右肺单肺叶切除病例352例(108例上叶与244例下叶)为对照组, 右肺双肺叶切除病例176例(57例中上叶与119例中下叶)为观察组。两组病例行回顾性病例-对照配对研究。两组病例按照年龄、性别、术前预计FEV1%、LEVF%、手术方式、术前心脏伴随疾病、术后管理方式、病理类型、肿瘤大小等情况进行按1:2进行配对。比较两组患者术后30天内死亡率、空间相关并发症(肺不张、漏气大于5天、气胸)发生率与心肺并发症(院内获得性肺炎、低氧气血症、肺栓塞、脑卒中、心律失常、心肌缺血或梗塞、心功能不全)发生率之间的差异。

**结果:**

双肺叶切除组病例术后30天死亡率为3.4%(6/176), 单肺叶切除组病例为2.3 %(8/352), 两者间差异无统计学意义; 双肺叶切除组病例术后心肺并发症总体发病率为23.8%(42/176)高于单肺叶切除组10.7%(38/352), 差异具有统计学意义; 双肺叶切除组中下叶切除病例的心肺并发症发病率为26.5%(31/119)远高于单肺叶切除组4.9%(12/244);而空间相关并发症双肺叶切除组与单肺叶切除组分别为20.4%(36/176)和17.3%(61/352), 两者间差异无统计学意义。

**结论:**

右侧NSCLC双肺叶切除患者术后心肺相关并发症发生率高于单肺叶切除患者, 30天内死亡率与空间相关并发症发生率无明显差异。

## Abstract (en)

**Background and objective:**

The mismatch between pleural space and remnant lung after bilobectomy has been considered as the main reason for the high incidence of postoperative complications in non-small cell lung cancer (NSCLC) patients.The aim of this study is to analyze the differences in postoperative complications between bilobectomy and lobectomy in the right lung of NSCLC patients.

**Method:**

This study included 528 NSCLC patients who underwent right pulmonary lobectomy.A total of 352 cases that underwent upper or lower right lobectomy (108 upper and 244 lower) were the control group, and 176 cases that underwent bilobectomy (57 upper and middle and 119 lower and middle) were the observation group.A retrospective case-control study was performed on a series of matched NSCLC patients.Cases and controls were matched by age, ppoFEV1%, LEVF%, operation method, cardiac comorbidity, type of postoperative management, and pathological type at a ratio of 1:2.The prevalence of 30-day death, occurrence of cardiac-respiratory complications (hospital-acquired pneumonia, low oxygen concentration, pulmonary embolism, cerebral apoplexy, arrhythmia, myocardial ischemia or infarction, and cardiac insufficiency) and occurrence of space-related complications (atelectasis, air leak more than 5 days, and pneumothorax) were compared between the bilobectomy and lobectomy groups.

**Result:**

The prevalence of 30-day death was 3.4% (6/176) in the bilobectomy group and 2.3% (8/352) in the lobectomy group.No statistical significance was observed between the two groups.The cardiac-respiratory complication rate in bilobectomy group (23.8%;42/176) was higher than that in lobectomy group (10.7%;38/352).The cardiac-respiratory complication rate of the lower and middle pulmonary lobectomy patients in the bilobectomy group (26.5%;31/119) was significantly higher than that in the lower pulmonary lobectomy patients (4.9%;12/244).The space-related complications in bilobectomy group and lobectomy group were 20.4% (36/176) and 17.3% (61/352), respectively.No statistically significant difference between the two groups was observed.

**Conclusion:**

The postoperative cardiac-respiratory complications of NSCLC patients with right bilobectomy are higher than those of the right lobectomy patients, but the prevalence of 30-day death and space-related complication was not statistically different between the two groups.

肺叶切除术是治疗非小细胞肺癌(non-small cell lung cancer, NSCLC)有效的治疗方法。NSCLC多发生于中、老年人, 患者术前常合并呼吸、循环等系统疾病, 接受肺叶切除术后, 肺实质组织减少, 肺通气功能不足, 造成缺氧, 肺淤血、肺水肿, 心脏及肺通气功能负荷增加, 使院内获得性肺炎、肺栓塞、脑卒中、心律失常、心肌缺血或梗死等心肺并发症发生率升高^[1, 2]^。切除单侧胸腔中一定数量的肺组织后, 剩余肺叶体积与胸膜腔容积不匹配, 胸膜腔内压力改变明显, 会使漏气时间延长(prolonged air leak, PAL)、肺不张、气胸等空间相关并发症发生率升高^[3]^。部分学者推荐行右肺双肺叶切除术可采取气腹术或其它措施, 以减少由于空间差异导致相关并发症的发生^[4, 5]^。

本研究对右肺单肺叶切除病例, 双肺叶切除病例行回顾性病例-对照配对研究, 比较两组患者术后空间相关并发症、心肺并发症以及术后死亡率之间的差异, 报道如下。

## 1 材料与方法

### 1.1 病例纳入标准与配对方法

本研究为病例对照配对回顾行研究, 病例选择为2005年3月-2012年6月昆明医科大学第三附属医院(云南肿瘤医院)胸外科一病区行右侧肺叶切除, 经病理确诊为右肺NSCLC病例526例。其中双肺叶切除病例176例(中上肺叶57例与中下肺叶119例)为观察组; 按1:2比例匹配单肺叶切除病例352例(上肺108例与下肺244例)为对照组。手术方式主要为传统右后侧开胸肺叶切除术与胸腔镜肺叶切除术(video-assisted thoracosopic surgery, VATS)。所有纳入病例没有标准化的预防漏气措施, 术后均留置单根胸腔引流管。无使用气腹法作为预防措施病例。病例的治疗模式多采用传统治疗模式, 部分病例采用胸外科快速康复(fast track surgery, FTS)治疗理念治疗^[6]^。本组研究中未选择扩大胸壁或膈膜切除病例, 全部纳入病例相关资料完整可溯源。

本研究分组为:右肺双肺叶切除病例(中上叶或中下叶切除)为观察组; 右肺单肺叶切除病例(上叶或下叶切除)为对照组。两组病例按照年龄、性别、术前预计FEV1%(ppoFEV1%)、左心室射血分数(LEVF%)、手术方式、管理模式、术前心脏风险评估、病理类型、肿瘤大小等情况进行按1:2进行配对。术前心脏伴随疾病(冠状动脉疾病、心脏手术史、高血压、充血性心力衰竭、需治疗的心律失常), 肿瘤大小以国际抗癌联盟(Union for International Cancer Control, UICC)2009年第七版肺癌TNM分期为pT分期标准。比较两组患者术后30天内死亡率、空间相关并发症与心肺并发症发生率之间的差异。

### 1.2 术后心肺并发症和空间并发症的评估标准

术后心肺相关并发症主要包括:院内获得性肺炎(使用美国疾控中心与医疗保健相关感染的监测定义医疗机构感染的分型标准)^[7]^; 呼吸道或通气不足(PO_2_ < 60 mmHg或PCO_2_ > 45 mmHg), 拔管后需再次行气管插管机械通气; 术后无需行外科介入处理的肺不张; 临床心功能不全; 肺栓塞; 心律失常; 心肌缺血或梗阻。

由于空间差异造成的空间并发症主要包括^[8]^:PAL(术后胸腔引流管持续漏气大于5天); 需要行支气管镜介入处理的肺不张; 需要外科穿刺排气的气胸, 无论是否存在胸腔引流管漏气。

### 1.3 统计方法

数据使用SPSS 17.0统计软件进行统计学分析, 采用配对资料卡方检验, *P* < 0.05为差异有统计学意义。

## 2 结果

经匹配后两组病例间年龄、性别、手术方式、肿瘤T分期、术前ppoFEV1%、LVEF%、心脏伴随疾病、病例治疗方式、术后病理类型等临床特征之间无明显差异(表 1)。

**1:** 双肺叶切除组与单肺叶切除组患者临床资料 Clinical characterizes of bilobectomy group and lobectomy group

Variable site	Bilobectomy group				Lobectomy group		
	Upper and middle (*n*=57)	Lower and middle(*n*=119)	Total (*n*=176)		Upper (*n*=108)	Lower (*n*=244)	Total (*n*=352)
Age, Mean (range)	64.3 (36.9-73.4)	63.1 (29.6-74.6)	63.4 (29.6-74.6)		62.6 (34.6-78.6)	61.5 (33.6-80.6)	61.8 (33.6-80.6)
ppoFEV1%, Mean (range)	57.9 (35.4-86.6)	56.2 (38.4-83.6)	56.6 (35.4-86.6)		55.8 (41.2-86.7)	54.6 (32.4-86.1)	55.4 (32.4-86.7)
LVEF%, Mean (range)	56.2 (49.2-64.4)	53.9 (46.2-59.4)	55.2 (46.2-64.4)		55.1 (48.2-58.2)	54.5 (49.4-61.3)	54.8 (48.2-61.3)
Gender, *n* (%)							
Male	25 (43.9)	62 (52.1)	87 (49.4)		58 (53.7)	132 (54.1)	190 (54.0)
Female	32 (56.1)	57 (47.9)	89 (50.6)		50 (46.2)	112 (45.9)	162 (46.0)
Pathological type, *n* (%)							
Squamous carcinoma	24 (42.1)	46 (38.6)	70 (40.0)		50 (46.2)	96 (39.3)	146 (41.5)
Adenocarcinoma	30 (52.6)	65 (54.6)	95 (54.0)		50 (48.1)	139 (57.0)	189 (53.7)
Other	3 (5.2)	8 (6.7)	11 (6.0)		8 (7.4)	9 (3.7)	17 (4.8)
Cardiac risk^a^, *n* (%)	11 (19.3)	34 (28.6)	45 (25.5)		16 (14.8)	72 (29.5)	88 (25.0)
pT1a-pT2a	46 (80.7)	93 (78.2)	139 (79.0)		76 (70.3)	186 (76.2)	262 (74.4)
FTS^b^	16 (28.1)	32 (26.9)	48 (27.2)		33 (30.6)	71 (29.1)	104 (29.5)
Surgical procedures, *n* (%)							
Thoracotomy	40 (70.2)	79 (66.4)	119 (67.6)		66 (61.1)	132 (54.1)	198 (56.2)
VATS	17 (29.8)	40 (33.6)	57 (32.4)		42 (38.9)	112 (45.9)	154 (43.8)
^a^Cases with cardiac comorbidity:coronary artery disease, previous cardiac surgery, arrhythmia, cardiac failure and hypertension; ^b^FTS:cases treated under a specific programme of fast track surgery.							

双肺叶切除组病例术后30天死亡率为:3.4%(6/176), 单肺叶切除组病例为:2.3%(8/352), 两者间差异无统计学意义; 双肺叶切除组病例术后心肺并发症发病率为23.8%(42/176)高于单肺叶组10.7 %(38/352), 差异具有统计学意义。而且在双肺叶切除组中下叶切除患者的心肺并发症发病率为26.5%(31/119), 远高于单肺叶组中下肺切除患者4.9%(12/244);而空间相关并发症双肺叶切除组与单肺叶切除组分别为20.4%(36/176)、17.3%(61/352), 两者间差异无统计学意义(表 2)。

**2:** 两组患者术后30天死亡率、心肺并发症发生率与空间相关并发症发生率 Prevalence of 30-d death, cardiorespiratory complications morbidity and space-related complications morbidity between two groups

Outcome	Site	Lobectomy group (%)	Bilobectomy group (%)
30-d death	All	8/352 (2.3)	6/176 (3.4)
	Upper	5/108 (4.6)	3/57 (5.3)
	Lower	3/244 (1.2)	3/119 (2.5)
Cardioresperatory morbidity	All	38/352 (10.7)	42/176 (23.8)^*^
	Upper	26/108 (24.0)	11/57 (19.2)
	Lower	12/244 (4.9)	31/119 (26.5)^**^
Space-related morbidity	All	61/352 (17.3)	36/176 (20.4)
	Upper	24/108 (22.2)	9/57 (15.7)
	Lower	37/244 (15.1)	25/119 (21.0)
^*^P < 0.01;^**^P < 0.01.			

## 3 讨论

肺叶切除是NSCLC的主要治疗方式之一, 对于肿瘤同时侵犯右肺中上叶或中下叶的患者而言, 双肺叶切除是其较好的治疗方式, 与单肺叶切除相比, 对患者远期生存率无明显影响^[9]^。手术方式、麻醉、术后管理措施等的差异均可对非小细胞肺癌患者术后并发症的发生造成影响。近年来研究^[1, 8, 9]^表明双肺叶切除患者术后并发症发病率较高, 接近50%。本研究双肺叶切除的患者术后并发症的发病率为44.1%, 低于相关报道结果, 可能是由于本组研究中部分病例采用FTS理念治疗, 使相关并发症发生率下降。

NSCLC患者双肺叶切除与单肺叶切除相比, 肺实质组织减少更多、剩余肺叶体积与胸膜腔容积差距更大。通常认为其心肺相关与空间相关并发症发病率高于单肺叶切除^[10]^, 但未见对双肺叶切除术后并发症发生率进行量化的相关报道。

本组研究采用病例对照配对法按1:2比例匹配单肺叶切除病例与双肺叶切除病例, 以排除年龄、心脏风险、切除肺叶部位、肿瘤大小导致的术后并发症的差异^[11, 12]^, 同时排除之前使用FTS治疗对患者术后并发症减少的影响。结果提示本组中双肺叶组30天死亡率3.4%稍高于单肺叶组2.3%, 本组病例中死亡病例14例, 心肺并发症6例, 其余病例死亡原因为:术后出血、重症感染以及肿瘤进展与是否双肺叶切除可能并无直接关系。双肺叶组病例术后心肺并发症总体发病率为23.8%, 高于单肺叶组10.7%;中下叶切除病例的心肺并发症发病率为26.5%明显高于下肺单叶切除的4.9%。提示NSCLC患者双肺叶切除后, 剩余肺实质不能提供足够的气体交换, 是导致单侧双肺切除术后患者心肺并发症的主要原因, 右肺中下叶切除患者, 术后更需警惕心肺相关并发症的出现。研究^[13]^表明:肺单叶切除术后5天-7天右心室后负荷增加, 舒张功能下降, 收缩功能无明显变化; 肺双叶切除术后5天-7天右心室后负荷增加, 舒张和收缩功能均下降, 心功能需约1月时间方能部分恢复。

对于空间相关并发症, 本组研究中提示双肺叶切除与单肺叶切除患者发生率分别为20.4%、17.3%, 两者间差异无统计学意义, 与理论中双肺叶切除后残余空间较大, 双肺叶切除患者空间相关并发症发生率应高于单肺叶切除的情况不符合^[1, 9]^。这可能是由于本研究中将空间相关并发症定义为剩余肺叶不能填满胸膜腔而带来的术后不良事件。由于缺乏胸膜腔的负压与术后的胸腔积气是导致残余肺组织局部受压, 是导致肺不张的第一步, 拔除胸管后出现气胸或因胸腔黏连气体不能经胸管排出, 本研究将需支气管镜介入处理的肺不张归类于空间相关并发症, 而对无需支气管镜介入处理的肺不张, 则划归为心肺相关并发症。这样的划归可能导致双肺叶切除并发症中肺不张比例下降。同时也与部分患者术前都有不同程度的肺气肿, 双肺肺叶切除后与单肺叶相比胸腔相对增大, 使得胸廓和剩余肺的顺应性明显升高相关; 部分患者由于肺癌肿瘤本身以及癌肿压迫所致肺不张以及痰液引流不畅刺激支气管产生痉挛, 导致原有肺组织局部受压, 当癌肿切除后, 被压缩的肺组织复张, 可部分抵消空间不匹配。双肺叶切除患者在术后1个月时通气功能可基本恢复至术前水平, 而肺部弥散功能则需3个月方能逐步恢复。

本研究未能证实NSCLC患者右肺双肺叶切除术后由于空间不匹配导致的空间并发症的发生率高于单肺叶切除, 但这仅是单个中心观察的结果, 可能需多中心的数据支持。NSCLC患者行双肺叶切除术后与心肺相关的并发症发生率高于单肺叶切除, 特别是右肺中下叶切除患者术后更需注意预防心肺相关并发症的发生。
